# Multigene phylogeny, phylogenetic network, and morphological characterizations reveal four new arthropod-associated *Simplicillium* species and their evolutional relationship

**DOI:** 10.3389/fmicb.2022.950773

**Published:** 2022-10-04

**Authors:** Wanhao Chen, Jiandong Liang, Xiuxiu Ren, Jiehong Zhao, Yanfeng Han, Zongqi Liang

**Affiliations:** ^1^Center for Mycomedicine Research, Basic Medical School, Guizhou University of Traditional Chinese Medicine, Guiyang, China; ^2^Institute of Fungus Resources, Department of Ecology, College of Life Sciences, Guizhou University, Guiyang, China

**Keywords:** spider, insect, multigene phylogeny, morphological characterization, phylogenetic relationship

## Abstract

*Simplicillium* species are widely distributed and commonly found on various substrates. A minority of species are associated with arthropods. A spider-associated species *Simplicillium araneae*, and three insect-associated species, *Simplicillium coleopterorum, Simplicillium guizhouense*, and *Simplicillium larvatum*, are proposed as novel species based on a multi-locus phylogenetic analysis and morphological characteristics. These *Simplicillium* species completely fit the nutritional model of Hypocreales fungi and could be used as a model to study their evolutionary relationship. A phylogenetic network analysis based on ITS sequences suggests that a host jump was common among *Simplicillium* species, and *S. araneae* may have originally come from an insect host and then jumped to a spider host. However, the evolutionary relationship of *S. coleopterorum, S. guizhouense*, and *S. larvatum* was not clear in the phylogenetic network and more sequencing information should be added to the network. In addition, strain CBS 101267 was identified as *Simplicillium subtropicum*.

## Introduction

The genus *Simplicillium* branched off from the genus *Verticillium* section *Prostrata*, and it consists of four species: *S. lanosoniveum* (J.F.H. Beyma) Zare and W. Gams, *S. obclavatum* (W. Gams) Zare and W. Gams, *S. lamellicola* (F.E.V. Sm.) Zare and W. Gams, and *S. wallacei* H.C. Evans (Zare and Gams, 2001). Zare and Gams (2001) summarized that solitary phialides, conidia adhering in globose, slimy heads or imbricate chains, and crystals commonly present in agar were the typical characteristics of *Simplicillium*. After that, numerous species were added to the genus (Liu and Cai, 2012; Nonaka et al., 2013; Gams, 2017; Zhang et al., 2017; Crous et al., 2018; Gomes et al., 2018; Chen et al., 2019, 2021; Wei et al., 2019; Kondo et al., 2020; Wang et al., 2020; Leplat et al., 2021). However, based mainly on rDNA sequence analyses, several *Simplicillium* species (*S. wallacei, S. coffeanum* A.A.M. Gomes and O.L. Pereira, *S. chinensis* F. Liu and L. Cai, and *S. filiforme* R.M.F. Silva, R.J.V. Oliveira, Souza-Motta, J.L. Bezerra and G.A. Silva) were transferred to the genera *Lecanicillium* W. Gams and Zare and *Leptobacillium* Zare and W. Gams (Zare and Gams, 2008; Okane et al., 2020; Chen et al., 2021). As a result, the genus *Simplicillium* currently consists of 23 species.

Chen et al. (2021) noted that *Simplicillium* species inhabit diverse substrates and could be used as a model of Hypocreales fungi to study their evolutional relationship. However, the phylogenetic tree assumes that biological groups evolve in the form of tree divergence and cannot accurately present the whole process of actual evolution, including hybridization, horizontal gene transfer, and gene recombination within the population (Cheng and Huang, 2008). The neighbor-net network (split network), a kind of distance-based phylogenetic network, can be used to present conflicting and ambiguous signals in datasets and detect subtle differences (Bryant and Moulton, 2004; Huson and Bryant, 2006). It can provide a way to present parallel events that are covered up and cannot be displayed by a phylogenetic tree, as well as an uncertain evolutionary phylogenetic relationship. The method has been applied in the phylogenetic analysis of animals, plants, and microorganisms (Bandelt and Dress, 1992; Morrison, 2005; Huson and Bryant, 2006; Morozov et al., 2013; Khonsanit et al., 2020).

During a survey of entomopathogenic fungi from Southwest China, some insect- and spider-associated specimens were found and some new *Simplicillium* strains were isolated and purified. The goals of this research were as follows: (1) identify the new strains based on ITS sequence, (2) characterize the new species of the genus *Simplicillium* based on a multi-locus phylogenetic analysis and their morphological and ecological characteristics, and (3) detect the evolutional relationship of the new species by the neighbor-net network based on ITS sequence of *Simplicillium* species.

## Materials and methods

### Specimen collection and identification

Five infected insect and spider specimens (DY1005, DY1025, DY10173, DY10181, and SD0538) were collected from Duyun City (26°21'24.71” N, 107°22'48.22” E) and Sandu County (25°57'22.21” N, 107°57'54.69” E), Guizhou Province, on 1 October and 1 May 2019. The surface of each insect body was rinsed with sterile water, followed by surface sterilization with 75% ethanol for 3–5 s and rinsing 3 times with sterilized water. After drying on sterilized filter paper, the synnemata, mycelium, or a part of the sclerotia was removed from the specimen, inoculated on potato dextrose agar (PDA), and improved potato dextrose agar (PDA, 1% w/v peptone) plates (Chen et al., 2019). Fungal colonies emerging from the specimens were isolated and cultured at 25°C for 14 days under 12 h light/12 h dark conditions following protocols described by Zou et al. (2010). The specimens and isolated strains were deposited at the Institute of Fungus Resources, Guizhou University (formally Herbarium of Guizhou Agricultural College; code, GZAC), Guiyang City, Guizhou, China.

Macroscopic characterization was determined from PDA cultures incubated at 25°C for 14 days, and the growth rate of the colony, the presence of octahedral crystals, and the colony colors (surface and reverse) were observed. To investigate the microscopic characteristics, a small number of mycelia were mounted in lactophenol cotton blue or 20% lactate acid solution and observed with an optical microscope (OM, DM4 B, Leica, Germany).

### DNA extraction, polymerase chain reaction amplification, and nucleotide sequencing

DNA extraction was carried out using a fungal genomic DNA extraction kit (DP2033, BioTeke Corporation) according to Liang et al. (2011). The extracted DNA was stored at −20°C. Amplification of the internal transcribed spacer (ITS) region, large subunit ribosomal RNA (LSU) gene, small subunit ribosomal RNA (SSU), RNA polymerase II largest subunit 1 (RPB1), and translation elongation factor 1 alpha (TEF) was carried out by PCR as described by White et al. (1990), Rakotonirainy et al. (1994), and Castlebury et al. (2004). Primer sequence information is shown in Supplementary Table S1. PCR products were purified and sequenced at Sangon Biotech (Shanghai) Co. The resulting sequences were submitted to GenBank (Table 1).

**Table 1:** Taxa included in the phylogenetic analyses.

**Species**	**Strain No**.	**GenBank accession No**.				
		**ITS**	**LSU**	**SSU**	**RPB1**	**TEF**
*Gamszarea wallacei*	CBS 101237	NR_111267	NG_042398	NG_062646	EF469102	EF469073
*Simplicillium album*	CGMCC 3.19635	NR_172844	NG_075278			MK336068
*Simplicillium aogashimaense*	JCM 18167	AB604002	LC496874	LC496889		LC496904
*Simplicillium aogashimaense*	JCM 18168	AB604004	LC496875	LC496890		
*Simplicillium araneae*	DY101811	OM743774	OM743792	OM743793		OM818465
*Simplicillium araneae*	DY101812	OM743840	OM743846	OM743845		OM818466
*Simplicillium calcicola*	LC5371	KU746705	KU746751			KX855251
*Simplicillium calcicola*	LC5586	KU746706	KU746752			KX855252
*Simplicillium cicadellidae*	GY11011	MN006243			MN022271	MN022263
*Simplicillium cicadellidae*	GY11012	MN006244			MN022272	MN022264
*Simplicillium coccinellidae*	DY101791	MT453861	MT453862	MT453863		MT471341
*Simplicillium coccinellidae*	DY101792	MT453864	MT457410			MT471342
*Simplicillium coleopterorum*	SD05381	OM743920	OM743925	OM743935		OM818467
*Simplicillium coleopterorum*	SD05382	OM744109	OM744170	OM744176		OM818468
*Simplicillium cylindrosporum*	JCM 18169	AB603989	LC496876	LC496891		LC496906
*Simplicillium cylindrosporum*	JCM 18170	AB603994	LC496877	LC496892		LC496907
*Simplicillium cylindrosporum*	JCM 18171	AB603997				
*Simplicillium cylindrosporum*	JCM 18172	AB603998				
*Simplicillium cylindrosporum*	JCM 18173	AB603999				
*Simplicillium cylindrosporum*	JCM 18174	AB604005				
*Simplicillium cylindrosporum*	JCM 18175	AB604006				
*Simplicillium formicae*	MFLUCC 18–1379	MK766511	MK766512	MK765046	MK882623	MK926451
*Simplicillium formicidae*	DL10041	MN006241			MN022269	
*Simplicillium formicidae*	DL10042	MN006242			MN022270	
*Simplicillium guizhouense*	DY10051	OM743225	OM743226	OM743242		OM818453
*Simplicillium guizhouense*	DY10052	OM743241	OM743252	OM743253		OM818454
*Simplicillium humicola*	CGMCC 3.19573	NR_172845	NG_075279			MK336071
*Simplicillium hymenopterorum*	DY101691	MT453848	MT453850	MT453849	MT471344	MT471337
*Simplicillium hymenopterorum*	DY101692	MT453851	MT453853	MT453852		MT471338
*Simplicillium lamellicola*	CBS 116.25	AJ292393	AF339552	AF339601	DQ522404	DQ522356
*Simplicillium lamellicola*	KYK00006	AB378533				
*Simplicillium lamellicola*	UAMH 2055	AF108471				
*Simplicillium lamellicola*	UAMH 4785	AF108480				
*Simplicillium lanosoniveum*	CBS 101267	AJ292395	AF339554		DQ522405	DQ522357
*Simplicillium lanosoniveum*	CBS 123.42	MH856100	MH867593			
*Simplicillium lanosoniveum*	CBS 704.86	AJ292396	AF339553	AF339602	DQ522406	DQ522358
*Simplicillium larvatum*	DY10251	OM743255	OM743351	OM743352	OM818455	OM818457
*Simplicillium larvatum*	DY10252	OM743431	OM743437	OM743436		OM818459
*Simplicillium larvatum*	DY101731	OM743438	OM743441	OM743453	OM818460	OM818462
*Simplicillium larvatum*	DY101732	OM743454	OM743485	OM743495		OM818464
*Simplicillium lepidopterorum*	GY29131	MN006246			MN022273	MN022265
*Simplicillium lepidopterorum*	GY29132	MN006245			MN022274	MN022266
*Simplicillium minatense*	JCM 18176	AB603992		LC496893		
*Simplicillium minatense*	JCM 18177	AB603991				
*Simplicillium minatense*	JCM 18178	AB603993		LC496894		
*Simplicillium neolepidopterorum*	DY101751	MT453854	MT453855	MT453856		MT471339
*Simplicillium neolepidopterorum*	DY101752	MT453857	MT453858	MT453859		MT471340
*Simplicillium niveum*	BCC 83036	MW621499	MW620992		MW603489	MW603488
*Simplicillium obclavatum*	CBS 311.74	AJ292394	AF339517	AF339567		EF468798
*Simplicillium obclavatum*	JCM 18179	AB604000				
*Simplicillium pechmerlense*	CBS 147188	MW031272	MW031268	MW031740	MW033222	MW033224
*Simplicillium scarabaeoidea*	DY101391	MT453842	MT453844	MT453843	MT471343	MT471335
*Simplicillium scarabaeoidea*	DY101392	MT453845	MT453846	MT453847		MT471336
*Simplicillium spumae*	JCM 39050	LC496869	LC496883	LC496898		LC496913
*Simplicillium spumae*	JCM 39051	LC496870	LC496884	LC496899		LC496914
*Simplicillium spumae*	JCM 39054	LC496871	LC496887	LC496902		LC496917
*Simplicillium subtropicum*	JCM 18180	AB603990	LC496880	LC496895		LC496910
*Simplicillium subtropicum*	JCM 18181	AB603995	LC496881	LC496896		LC496911
*Simplicillium subtropicum*	JCM 18182	AB603996				
*Simplicillium subtropicum*	JCM 18183	AB604001				
*Simplicillium sympodiophorum*	JCM 18184	AB604003	LC496882	LC496897		LC496912
*Simplicillium yunnanense*	YFCC 7133		MN576784	MN576728	MN576844	MN576954
*Simplicillium yunnanense*	YFCC 7134		MN576785	MN576729	MN576845	MN576955

### Sequence alignment and phylogenetic and network analyses

Lasergene software (version 6.0, DNASTAR) was used to edit DNA sequences in this study. The SSU, ITS, LSU, RPB1, and TEF sequences were downloaded from GenBank, based on Nonaka et al. (2013), Zhang et al. (2017), Crous et al. (2018, 2021), Gomes et al. (2018), Chen et al. (2019, 2021), Wei et al. (2019), Kondo et al. (2020), Leplat et al. (2021), and others selected based on BLAST searches in GenBank (Table 1). ITS sequence was applied to identify the new strains in the genus *Simplicillium* (analysis 1). ITS sequences and other loci were aligned and edited by MAFFT v7.037b (Katoh and Standley, 2013) and MEGA6 (Tamura et al., 2013). Combined sequences of SSU, ITS, LSU, RPB1, and TEF were applied to establish the four novel species (analysis 2) and obtained using SequenceMatrix v.1.7.8 (Vaidya et al., 2011). The model was selected for Bayesian analysis by ModelFinder (Kalyaanamoorthy et al., 2017) in PhyloSuite software Zhang et al. (2020a).

ITS sequences and the combined loci were analyzed using Bayesian inference (BI) and maximum likelihood (ML) methods. For BI, a Markov chain Monte Carlo (MCMC) algorithm was used to generate phylogenetic trees with Bayesian probabilities using MrBayes v.3.2 (Ronquist et al., 2012) for the combined sequence datasets. The Bayesian analysis resulted in 20,001 trees after 10,000,000 generations. The first 4,000 trees, representing the burn-in phase of the analysis, were discarded, while the remaining 16,001 trees were used to calculate posterior probabilities in the majority rule consensus tree. After the analysis was finished, each run was examined using the program Tracer v1.5 (Drummond and Rambaut, 2007) to determine burn-in and confirm that both runs had converged. ML analyses were constructed with RAxMLGUI (Silvestro and Michalak, 2012). The GTRGAMMA model was used for all partitions, in accordance with recommendations in the RAxML manual against the use of invariant sites. The phylogenetic network was constructed by SplitsTree 4 using the neighbor-net method based on the ITS sequence, which was the most common sequence in the genus *Simplicillium*. The other condition was the default settings (Huson and Bryant, 2006).

## Results

### Phylogenetic and network analyses

**Figure 1:**
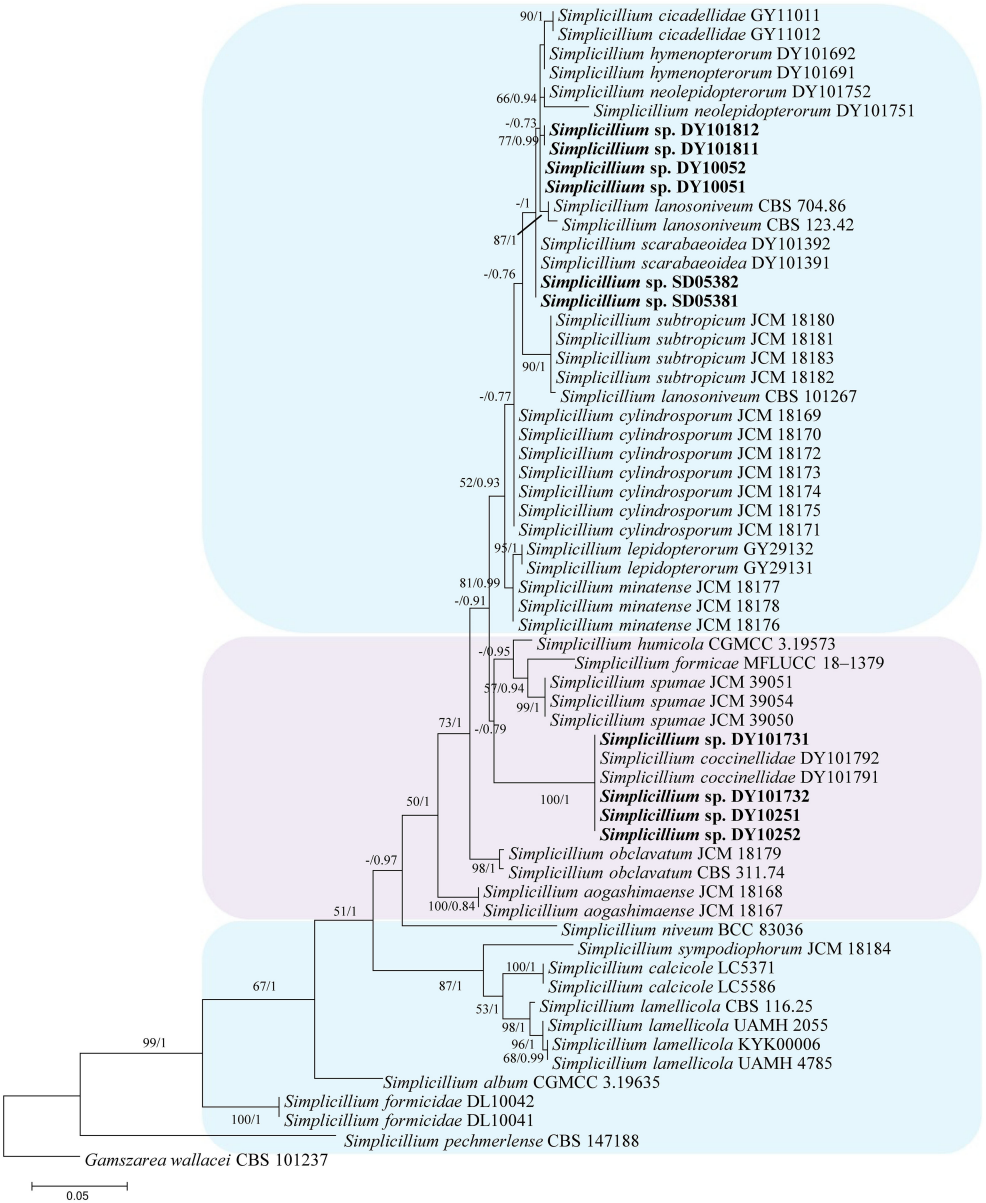
Phylogenetic identification of the new strains in the genus *Simplicillium* based on ITS sequence. Statistical support values (≥ 0.5/50%) are shown at the nodes for ML bootstrap support/BI posterior probabilities.

**Figure 2:**
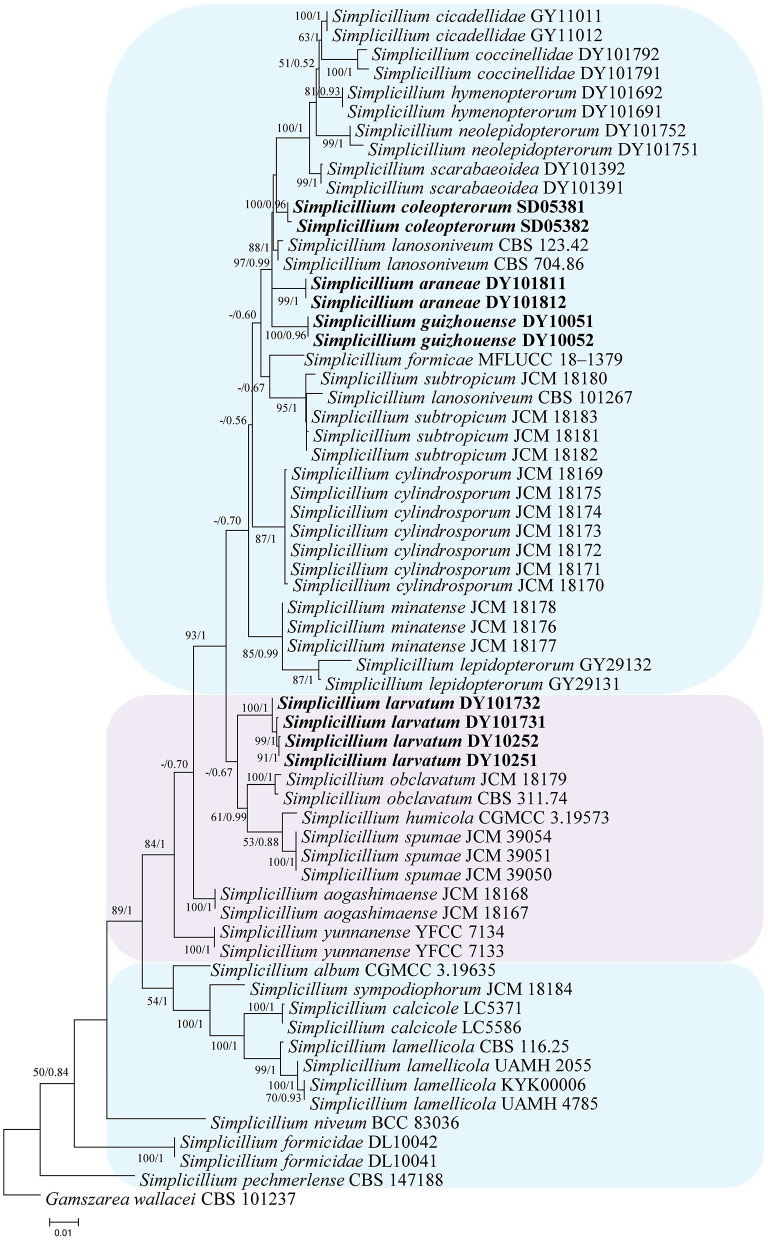
Phylogenetic analysis to establish the new species in the genus *Simplicillium* by SSU, ITS, LSU, RPB1, and TEF sequences. Statistical support values (≥ 0.5/50%) are shown at the nodes for ML bootstrap support/BI posterior probabilities.

In the phylogenetic tree of analysis 1 (to identify new strains in the genus *Simplicillium*) and analysis 2 (to establish four novel species) (Figures 1, 2, respectively), *Gamszarea wallacei* (H.C. Evans) Z.F. Zhang and L. Cai (CBS 101237) was used as the outgroup. The ITS sequence and concatenated sequences (SSU, ITS, LSU, RPB1, and TEF) of analyses 1 and 2 included 23 and 24 taxa, respectively, and consisted of 547 (ITS) and 3,544 (SSU, 982; ITS, 547; LSU, 624; RPB1, 544; and TEF, 847) characters with gaps, respectively.

Analysis 1: The final value of the highest scoring tree was −2,850.268342, which was obtained from an ML analysis of the ITS sequence. The parameters of the General Time Reversible (GTR) model used to analyze the dataset were estimated using the following frequencies: A = 0.230169, C = 0.278999, G = 0.261573, and T = 0.229259; substitution rates AC = 1.092678, AG = 1.861964, AT = 1.258018, CG = 0.827521, CT = 3.497952, and GT = 1.000000, as well as the gamma distribution shape parameter α = 0.302260. The selected model for BI analysis was K2P+G4 (ITS). The phylogenetic trees (Figure 1) constructed using ML and BI analyses were largely congruent and strongly supported in most branches. Strains DY10051, DY10052, DY101811, and DY101812 were grouped with *Simplicillium cicadellidae* W.H. Chen, C. Liu, Y.F. Han, J.D. Liang and Z.Q. Liang, *S. hymenopterorum* W.H. Chen et al., *S. lanosoniveum*, and *S. neolepidopterorum* W.H. Chen et al. Strains SD05381 and SD05382 were grouped with *S. scarabaeoidea* W.H. Chen et al. Strains DY10251, DY10252, DY101731, and DY101732 have a close relationship with *S. coccinellidae* W.H. Chen et al.

Analysis 2: The final value of the highest scoring tree was −17,350.543656, which was obtained from the ML analysis of the dataset (SSU+ITS+LSU+RPB1+TEF). The parameters of the GTR model used to analyze the dataset were estimated based on the following frequencies, A = 0.242448, C = 0.266436, G = 0.262106, and T = 0.229010; substitution rates AC = 1.080009, AG = 1.915942, AT = 1.147141, CG = 0.835034, CT = 5.341508, and GT = 1.000000, as well as the gamma distribution shape parameter α = 0.291522. The selected model for BI analysis was K2P+G4 (LSU) and SYM+I+G4 (SSU+ITS+RPB1+TEF). The phylogenetic trees (Figure 2) constructed using ML and BI analyses were largely congruent and strongly supported in most branches. Most genera were clustered into their independent clade. The new strains were clustered into four independent clades. *Simplicillium larvatum* (DY101731, DY101732, DY10251, and DY10252) had a close relationship with *S. obclavatum, S. humicola* Z.F. Zhang and L. Cai, and *S. spumae* N. Kondo, H. Iwasaki and Nonaka. *S. araneae* (DY101811 and DY101812), *S. guizhouense* (DY1005 and DY10052), and *S. coleopterorum* (SD05381 and SD05382) had a close relationship with *S. lanosoniveum*.

**Figure 3:**
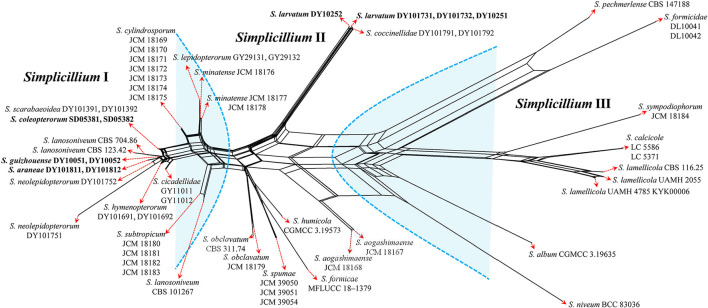
Reconstruction of the neighbor-net network based on ITS sequences from taxa in Figure 1.

The topological structure of the network is consistent with that of the phylogenetic tree (Figure 1) and could be used for species relationship analysis (Huson and Bryant, 2006). However, a reticular structure was formed in the phylogenetic network by the split of information conflict or fuzzy signals. Three groups were present in the phylogenetic network (Figure 3).

Group I: New strains DY10051, DY10052, DY101811, DY101812, SD05381, and SD05382 grouped with *Simplicillium cicadellidae* (GY11011 and GY11012), *S. cylindrosporum* Nonaka, Kaifuchi and Masuma (JCM 18169, JCM 18170, JCM 18171, JCM 18172, JCM 18173, JCM 18174, and JCM 18175), *S. hymenopterorum* (DY101691 and DY101692), *S. lanosoniveum* (CBS 101267, CBS 123.42, and CBS 704.86), *S. lepidopterorum* W.H. Chen et al. (GY29131 and GY29132), *S. minatense* Nonaka, Kaifuchi and Masuma (JCM 18176, JCM 18177, and JCM 18178), *S. neolepidopterorum* (DY101751 and DY101752), and *S. scarabaeoidea* (DY101391 and DY101392).

Group II: *S. aogashimaense* Nonaka, Kaifuchi and Masuma (JCM 18167 and JCM 18168) grouped with *S. formicae* D.P. Wei and K.D. Hyde (MFLUCC 18–1379), *S. humicola* (CGMCC 3.19573), *S. obclavatum* (CBS 311.74 and JCM 18179), and *S. spumae* (JCM 39050, JCM 39051 and JCM 39054). The new strains DY10251, DY10252, DY101731 and DY101732 are clustered with *S. coccinellidae* (DY101791 and DY101792) into an independent subgroup.

Group III: *S. album* Z.F. Zhang and L. Cai (CGMCC 3.19635) grouped with *S. calcicole* Z.F. Zhang, F. Liu and L. Cai (LC5371 and LC5586), *S. formicidae* W.H. Chen et al. (DL10041 and DL10042), *S. lamellicola* (CBS 116.25, KYK00006, UAMH 2055 and UAMH 4785), *S. niveum* Mongkols., Noisrip. and Luangsa-ard (BCC 83036), *S. pechmerlense* J. Leplat (CBS 147188), and *S. sympodiophorum* Nonaka, Kaifuchi and Masuma (JCM 18184).

## Taxonomy

### *Simplicillium araneae* W.H. Chen, Y.F. Han, J.D. Liang and Z.Q. Liang, sp. nov.

MycoBank: 844146.

Type: CHINA, Guizhou, Qiannan Buyi, and Miao Autonomous Prefecture, Duyun City (26°21'27.96”N, 107°22'48.22”E). On a dead spider (Araneae), 1 October 2019, Wanhao Chen, GZAC DY10181 (holotype), ex-type living cultures, DY101811.

Description: The colonies showed moderate growth on PDA, reaching a diameter of 31–33 mm in 14 days at 25°C, were convex, with white velutinate aerial mycelium on the front and an yellowish to brown mycelium on the reverse, especially in the middle and entire margins, and soluble pigment was not produced. Vegetative hyphae were branched, hyaline, smooth-walled, septate, and 1.2–1.8 μm wide. The phialides produced on the aerial hyphae were always solitary, aseptate, hyaline, smooth-walled, relatively slender, tapering toward the tip, and 32.9–47.1 × 1.2–2.4 μm in size. Conidia hyaline was ellipsoidal to globose, aseptate, smooth-walled, 1-celled, and 1.8–2.9 × 1.2–1.8 μm in size. Octahedral crystals were absent, and a sexual state was not observed.

Etymology. Referring to the ability to colonize spiders.

Additional strain examined. China, Guizhou, Qiannan Buyi, and Miao Autonomous Prefecture, Duyun City (26°21'27.96”N, 107°22'48.22”E). On a dead spider (Araneae), 1 October 2019, Wanhao Chen, DY101812.

**Figure 4:**
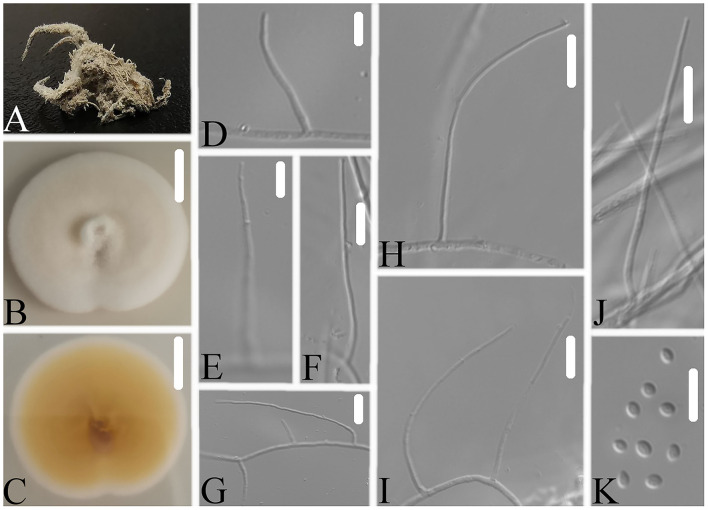
*Simplicillium araneae* **(A)** Infected spider (Araneae); **(B,C)** PDA-containing culture plate showing the front **(B)** and reverse **(C)** sides of the colony; **(D–K)** solitary phialides and conidia. Scale bars: 10 mm **(B,C)** and 10 μm **(D–K)**.

Notes: *Simplicillium araneae* was identified as belonging to *Simplicillium* because of its solitary phialides (Figure 4) and the analysis ITS sequence (Figure 1). Compared to the typical characteristics of 23 species, *S. araneae* is morphologically similar to *S. formicae, S. hymenopterorum*, and *S. neolepidopterorum* based on the absence of a slime head and octahedral crystals. However, based on the combined dataset of SSU, ITS, LSU, RPB1, and TEF sequences (Figure 2), *S. araneae* clustered into an independent clade and was distinguished from other *Simplicillium* species.

### *Simplicillium coleopterorum* W.H. Chen, Y.F. Han, J.D. Liang, and Z.Q. Liang, sp. nov.

MycoBank: 844147.

Type: CHINA, Guizhou, Qiannan Buyi, and Miao Autonomous Prefecture, Sandu County (25°57'22.21” N, 107°57'54.69” E). On a beetle (Coleoptera), 1 May 2019, Wanhao Chen, GZAC SD0538 (holotype), ex-type living cultures, SD05381.

Description: The colonies showed moderate growth on PDA, reaching a diameter of 49–50 mm in 14 days at 25°C, and convex, with white velutinate aerial mycelium on the front and pale brown to brown mycelium on the reverse, especially in the middle and entire margins, and soluble pigment was not produced. The vegetative hyphae were branched, hyaline, smooth-walled, septate, and 1.0–1.6 μm wide. The phialides produced on the aerial hyphae were always solitary, aseptate, hyaline, smooth-walled, relatively slender, tapering toward the tip, and 34.5–64.1 × 0.7–1.2 μm in size. Conidia were observed as small sub-globose slimy heads at the apex of the phialides, hyaline, ellipsoidal to globose in shape, aseptate, smooth-walled and 1-celled, and 2.1–3.3 × 1.5–1.9 μm in size. Octahedral crystals were absent, and a sexual state was not observed.

Etymology: Referring to an insect host in order Coleoptera.

Additional strain examined: China, Guizhou, Qiannan Buyi and Miao Autonomous Prefecture, Sandu County (25°57'22.21” N, 107°57'54.69” E). On a beetle (Coleoptera), 1 May 2019, Wanhao Chen, SD05382.

**Figure 5:**
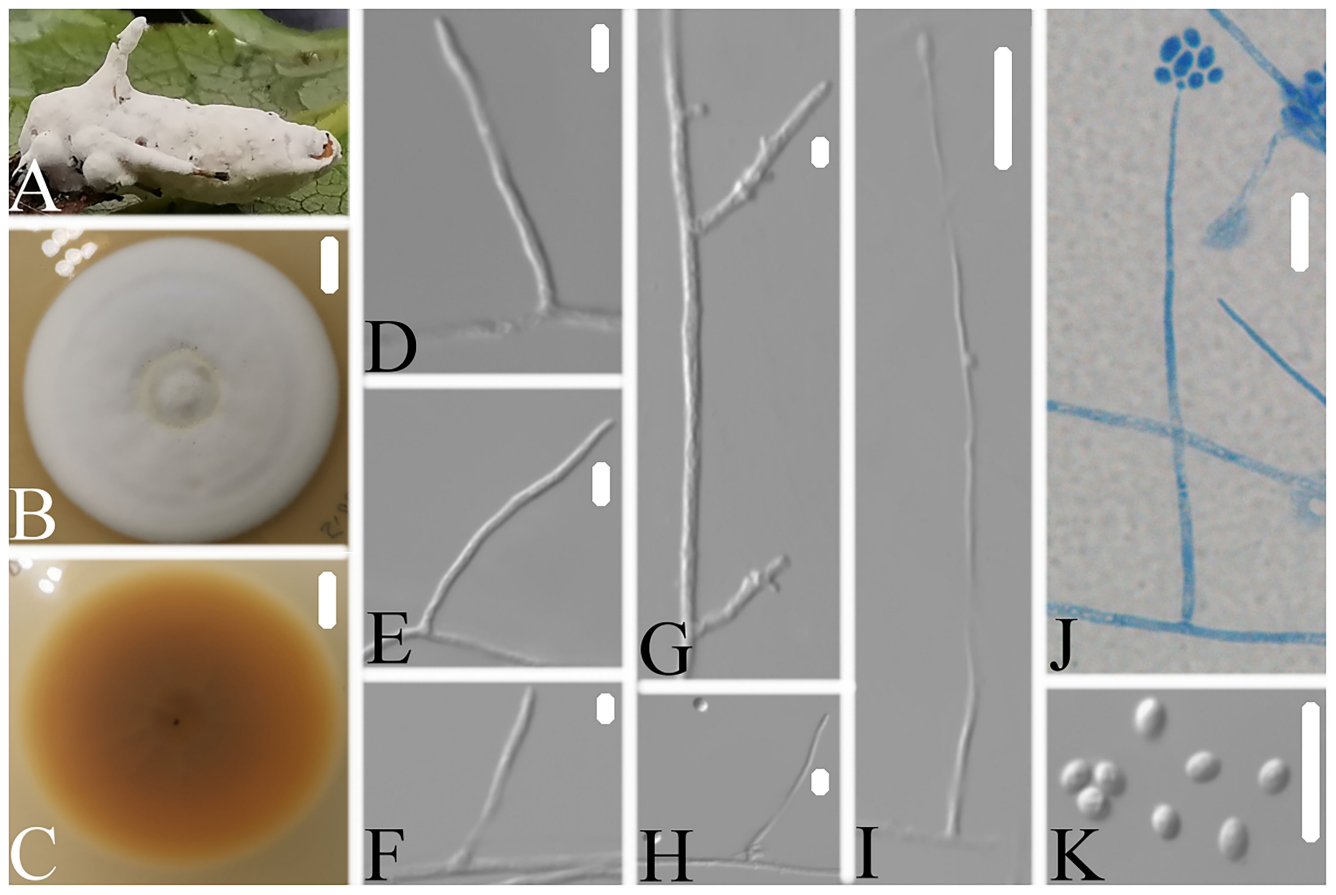
*Simplicillium coleopterorum* **(A)** Infected insect (Coleoptera); **(B,C)** PDA-containing culture plate showing the front **(B)** and reverse **(C)** sides of the colony; **(D–K)** solitary phialides and conidia. Scale bars: 10 mm **(B,C)** and 10 μm **(D–K)**.

**Figure 6:**
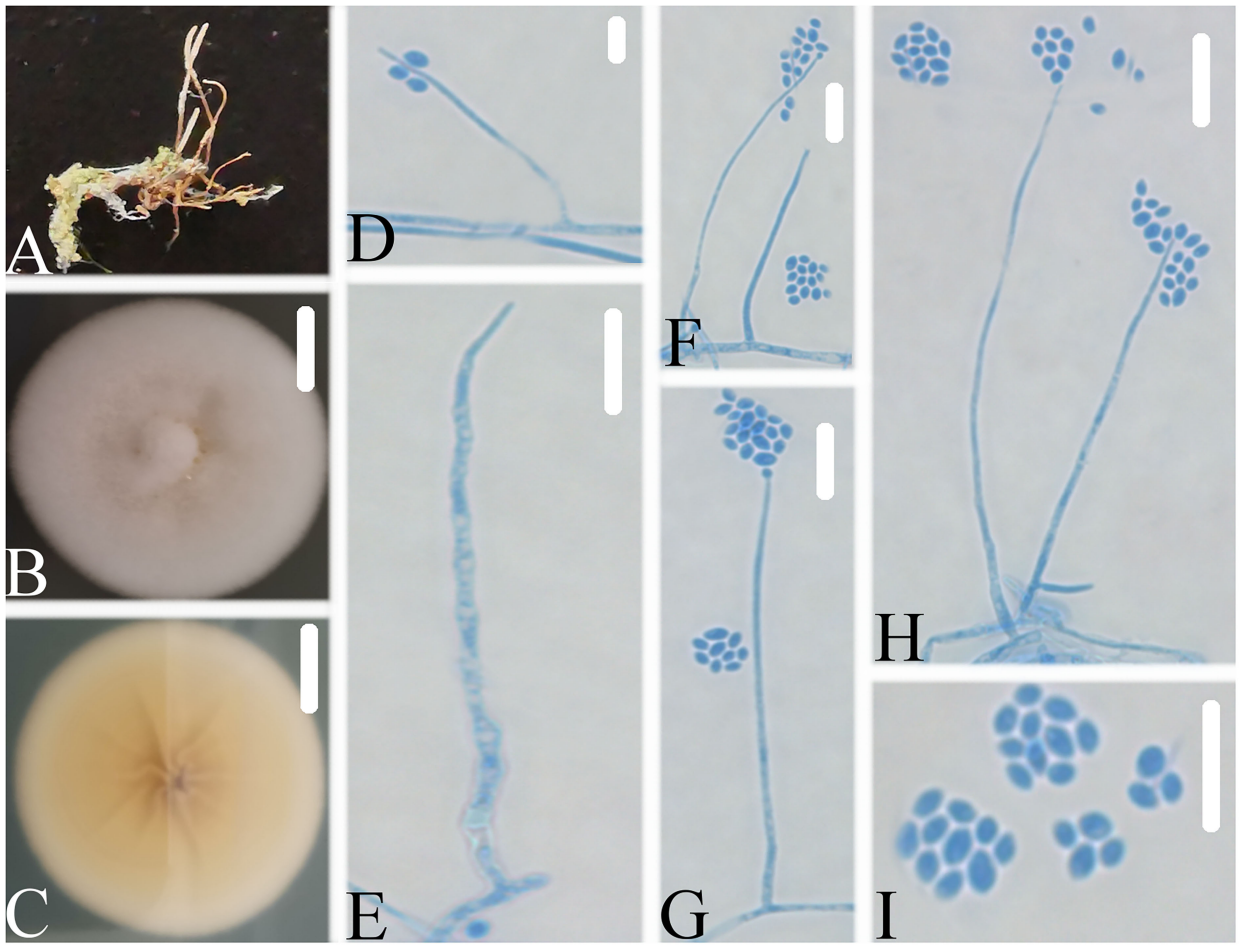
*Simplicillium guizhouense* **(A)** Infected ant (Formicidae); **(B,C)** PDA-containing culture plate showing the front **(B)** and reverse **(C)** sides of the colony; **(D–I)** solitary phialides and conidia. Scale bars: 10 mm **(B,C)** and 10 μm **(D–I)**.

Notes: *Simplicillium coleopterorum* was identified as belonging to *Simplicillium* because of its solitary phialides, conidia adhering in subglobose slimy heads, and the absence of octahedral crystals (Figure 5), supported by phylogenetic analysis of ITS sequence (Figure 1). Compared with the typical characteristics of 23 species, *S. coleopterorum* was morphologically similar to *S. cicadellidae, S. coccinellidae, S. formicidae, S. lepidopterorum, S. niveum, S. scarabaeoidea*, and *S. yunnanense* (Figure 6). However, based on the combined dataset of SSU, ITS, LSU, RPB1, and TEF sequences (Figure 2), *S. coleopterorum* was clustered into an independent clade and distinguished from *S. cicadellidae, S. coccinellidae, S. formicidae, S. lepidopterorum, S. niveum, S. scarabaeoidea*, and *S. yunnanense*.

### *Simplicillium guizhouense* W.H. Chen, Y.F. Han, J.D. Liang and Z.Q. Liang sp. nov.

MycoBank: 844148.

Type: CHINA, Guizhou, Qiannan Buyi and Miao Autonomous Prefecture, Duyun City (26°21'27.96”N, 107°22'48.22”E). On an ant (Formicidae), 1 October 2019, Wanhao Chen, GZAC DY1005 (holotype), ex-type living cultures, DY10051.

Description: The colonies showed moderate growth on PDA, reaching a diameter of 35–36 mm in 14 days at 25°C, and were convex, with white velutinate aerial mycelium on the front and yellowish to pale yellowish mycelium on the reverse, especially in the middle and entire margin, and soluble pigment was not produced. The vegetative hyphae were branched, hyaline, smooth-walled, septate, and 1.4–1.5 μm wide. The phialides produced on the aerial hyphae were always solitary, aseptate, hyaline, smooth-walled, relatively slender, and tapering toward the tip, and 21.1–52.2 × 1.0–1.8 μm in size. Conidia were observed as small globose slimy heads at the apex of the phialides, hyaline, ellipsoidal in shape, aseptate, smooth-walled and 1-celled, and 2.4–2.9 × 1.6–1.8 μm in size. Octahedral crystals were absent, and a sexual state was not observed.

Etymology: Referring to the place where the fungus was collected.

Additional strain examined: China, Guizhou, Qiannan Buyi and Miao Autonomous Prefecture, Duyun City (26°21'27.96”N, 107°22'48.22”E). On an ant (Formicidae), 1 October 2019, Wanhao Chen, DY10052.

Notes: *Simplicillium guizhouense* is morphologically similar to *S. cicadellidae, S. coccinellidae, S. formicidae, S. lepidopterorum, S. niveum, S. scarabaeoidea*, and *S. yunnanense* (Figure 6). Based on the combined dataset of SSU, ITS, LSU, RPB1, and TEF sequences (Figure 2), *S. guizhouense* clustered into an independent clade, and was distinguished from *S. cicadellidae, S. coccinellidae, S. formicidae, S. lepidopterorum, S. niveum, S. scarabaeoidea*, and *S. yunnanense*.

### *Simplicillium larvatum* W.H. Chen, Y.F. Han, J.D. Liang and Z.Q. Liang, sp. nov.

MycoBank: 844149.

Type: CHINA, Guizhou, Qiannan Buyi and Miao Autonomous Prefecture, Duyun City (26°21'27.96”N, 107°22'48.22”E). On a larva (Lepidoptera), 1 October 2019, Wanhao Chen, GZAC DY10173 (holotype), ex-type living cultures, DY101731.

Description: The colonies showed moderate growth on PDA, reaching a diameter of 40–43 mm in 14 days at 25°C, and were convex, with white velutinate aerial mycelium on the front and yellowish mycelium on the reverse, especially in the middle and entire margin, and soluble pigment was not produced. The vegetative hyphae were branched, hyaline, smooth-walled, septate, and 0.9–1.6 μm wide. The phialides produced on the aerial hyphae were always solitary, aseptate, hyaline, smooth-walled, relatively slender, tapering toward the tip, and 16.4–28.7 × 1.2–1.7 μm in size. Conidia were observed as small sub-globose slimy heads at the apex of the phialides, hyaline, ellipsoidal to long ellipsoidal in shape, aseptate, smooth-walled and 1-celled, and 1.8–3.3 × 1.6–2.0 μm in size. Octahedral crystals were absent, and a sexual state was not observed.

Etymology: Referring to its insect host, a larva.

Additional strain and specimen examined. China, Guizhou, Qiannan Buyi and Miao Autonomous Prefecture, Duyun City (26°21'27.96”N, 107°22'48.22”E). On a larva (Lepidoptera), 1 October 2019, Wanhao Chen, DY101732; on a pupa (Lepidoptera), 1 October 2019, Wanhao Chen, GZAC DY1025, living cultures, DY10251, DY10252.

**Figure 7:**
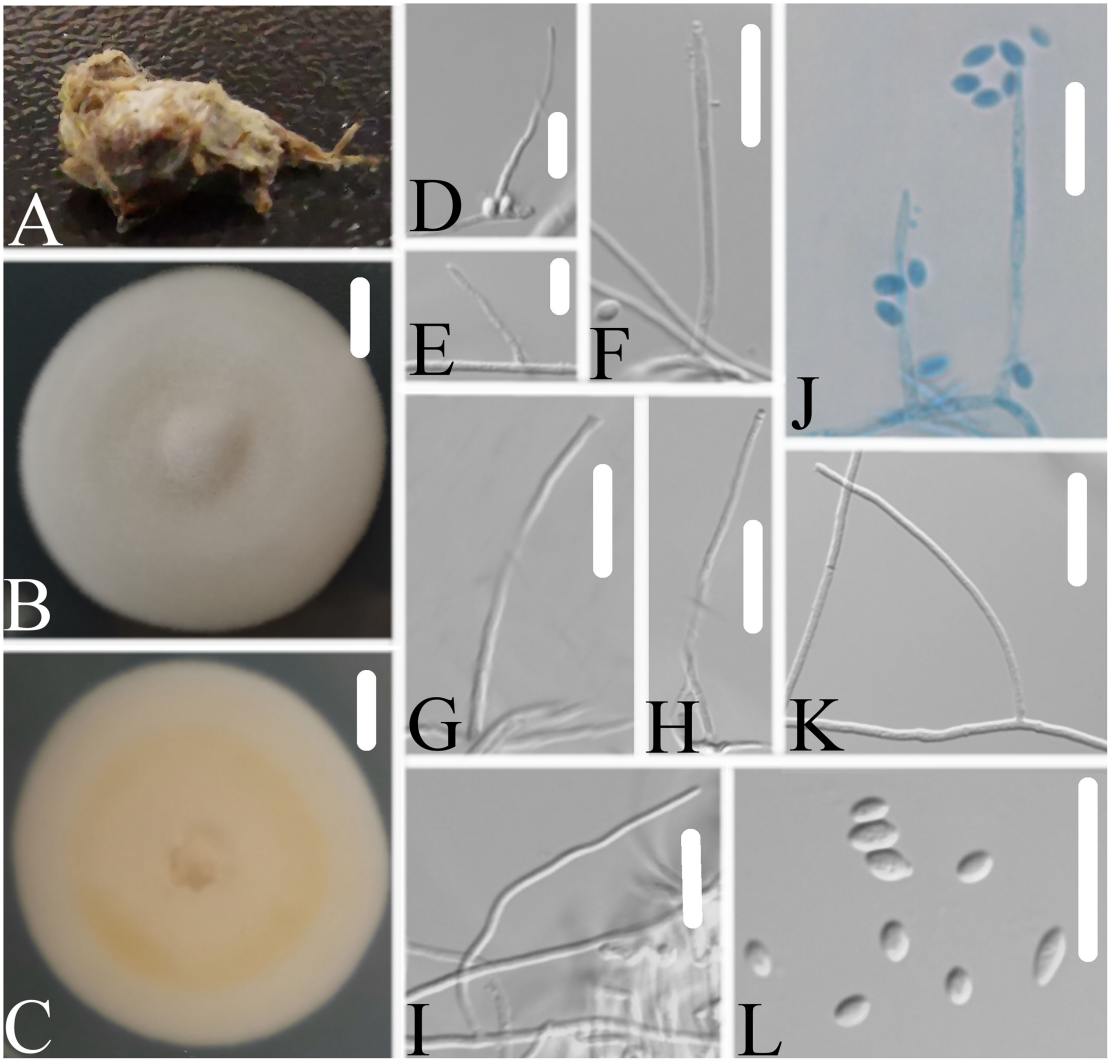
*Simplicillium larvatum* **(A)** Infected larva (Lepidoptera); **(B,C)** PDA-containing culture plate showing the front **(B)** and reverse **(C)** sides of the colony; **(D–L)** solitary phialides and conidia. Scale bars: 10 mm **(B,C)** and 10 μm **(D–L)**.

Notes: *Simplicillium larvatum* is morphologically similar to *S. cicadellidae, S. coccinellidae, S. formicidae, S. lepidopterorum, S. niveum, S. scarabaeoidea*, and *S. yunnanense* (Figure 7). However, based on the combined dataset of SSU, ITS, LSU, RPB1, and TEF sequences (Figure 2), *S. larvatum* was clustered into an independent clade and phylogenetically close to *S. humicola, S. obclavatum*, and *S. spumae*. However, *S. larvatum* was morphologically distinguished from *S. humicola*, which has bigger conidia (3.0–5.0 × 1.5–3.0 μm) with octahedral crystals present. *S. larvatum* was morphologically distinguished from *S. obclavatum*, which has longer phialide (30–52 × 0.8–2.0 μm) with octahedral crystals present. *S. larvatum* was morphologically distinguished from *S. spumae*, which has subglobose or oval to ellipsoidal and octahedral crystals present.

## Discussion

Zare and Gams (2001) noted that *Simplicillium* species are widely distributed and commonly found on various substrates or as fungicolous fungi. *S. album, S. calcicola, S. cylindrosporum, S. humicola, S. minatense, S. obclavatum, S. pechmerlense, S. subtropicum*, and *S. sympodiophorum* were isolated from soil, marine water, rock, decaying wood, and air (Zare and Gams, 2001; Liu and Cai, 2012; Nonaka et al., 2013; Liang et al., 2017; Zhang et al., 2020b; Leplat et al., 2021). *S. aogashimaense* was isolated from the soil and has also been reported as a symbiotic fungus (Nonaka et al., 2013; Shentu et al., 2020). *S. lanosoniveum* was reported as both an endophytic and an hyperparasitic fungus (Baiswar et al., 2014; Wei et al., 2019). *S. formicae, S. lamellicola, S. niveum*, and *S. yunnanense* were reported as hyperparasitic fungi (Shin et al., 2017; Wei et al., 2019; Wang et al., 2020; Crous et al., 2021). *S. cicadellidae, S. coccinellidae, S. formicidae, S. hymenopterorum, S. lepidopterorum, S. neolepidopterorum*, and *S. scarabaeoidea* were reported to be associated with different insects (Chen et al., 2019, 2021). In the present study, four new species associated with spiders and other insect substrates have been reported. Thus, *Simplicillium* species completely fit with the nutritional model of Hypocreales fungi and may be used as a model to study their evolutionary relationship.

In the present study, the phylogenetic network was reconstructed to explore the evolutionary relationship, consistent with the phylogenetic tree of the ITS sequence and the combined datasets (SSU, ITS, LSU, RPB1, TEF). According to the phylogenetic network (Figure 3), *Simplicillium guizhouense* (DY10051 and DY10052) may share a common ancestor with *S. araneae* (DY101811 and DY101812), *S. lanosoniveum* (CBS 123.42 and CBS 704.86), and *S. neolepidopterorum* (DY101751 and DY101752). *S. lepidopterorum* (GY29131 and GY29132) may share a common ancestor with *S. minatense* (JCM 18176, JCM 18177, and JCM 18178). *S. obclavatum* (CBS 311.74 and JCM 18179) may share a common ancestor with *S. formicae* (MFLUCC 18–1379), *S. humicola* (CGMCC 3.19573), and *S. spumae* (JCM 39050, JCM 39051 and JCM 39054). *S. calcicole* (LC5371 and LC5586) may share a common ancestor with *S. lamellicola* (CBS 116.25, UAMH 2055, KYK00006, UAMH 4785).

Host shift is usually described as an evolutional process between fungi and their hosts and is often determined by nutrient requirements (Vega et al., 2009). The nutritional model of Hypocreales fungi goes from plants (including living plants and plant residues) to animals (especially insects) and finally to fungi (Spatafora et al., 2007). The results of the phylogenetic network suggest that *S. araneae, S. lanosoniveum*, and *S. neolepidopterorum* may have originally come from insects and then jumped to a spider host, plant and fungi substrate, or another insect host, respectively. *S. lepidopterorum* may have originally come from the soil and then jumped to an insect host. *S. formicae* and *S. spumae* may have originally come from air or soil and then jumped as hyperparasitic fungi or water substrates. *S. lamellicola* may have originally come from rock substrate and then jumped as hyperparasitic fungus. These results suggest that host jump may be common in *Simplicillium* species.

*S. coleopterorum* and *S. larvatum* could not split from *S. scarabaeoidea* and *S. coccinellidae* in the phylogenetic network as only the ITS sequence was analyzed. However, they could be phylogenetically distinguished by a multi-locus phylogenetic analysis. Therefore, more sequence information should be added to the phylogenetic network in order to analyze their evolutionary relationship. Moreover, *S. lanosoniveum* was transferred to the genus *Simplicillium* by Zare and Gams (2001) with the strain CBS 123.42. In the present study, three strains of *S. lanosoniveum* (CBS 101267, CBS 123.42, and CBS 704.86) were tested. Strains CBS 123.42 and CBS 704.86 were clustered into a subclade. However, strain CBS 101267 was clustered with four strains of *S. subtropicum* (JCM 18180, JCM 18181, JCM18182, and JCM 18183). The pairwise dissimilarity of ITS sequences shows only a 4 bp difference, with 552 bp between strains CBS 101267 and JCM18180. This result supports strain CBS 101267 being identified as *S. subtropicum*.

## Data availability statement

The original contributions presented in the study are included in the article/Supplementary material, further inquiries can be directed to the corresponding author.

## Author contributions

WC isolated the fungi, built up the phylogenetic tree, and wrote the manuscript. YH identified the fungal isolates, revised the manuscript, and provided partial funding. JL, XR, and JZ revised the manuscript and provided partial funding. ZL identified the fungal isolates and revised the manuscript. All authors discussed the results.

## Funding

This work was supported by the National Natural Science Foundation of China (Grant No. 31860002), the High-level Innovative Talents Training Object in Guizhou Province (No. Qiankehepingtairencai [2020]6005), the Science and Technology Foundation of Guizhou Province (No. Qiankehejichu [2020]1Y060), the Program of Innovative Scientific and Technological Talent Team of Guizhou Province (2020-5010), and the Construction Program of Guizhou Engineering Research Center (Qian Fa Gai Gao Ji 2020-896).

## Conflict of interest

The authors declare that the research was conducted in the absence of any commercial or financial relationships that could be construed as a potential conflict of interest.

## Publisher's note

All claims expressed in this article are solely those of the authors and do not necessarily represent those of their affiliated organizations, or those of the publisher, the editors and the reviewers. Any product that may be evaluated in this article, or claim that may be made by its manufacturer, is not guaranteed or endorsed by the publisher.
