# Supplementary material for: Multigene phylogeny, phylogenetic network, and morphological characterizations reveal four new arthropod-associated Simplicillium species and their evolutional relationship
Source: Front Microbiol. 2022 Oct 4;13:950773. doi: 10.3389/fmicb.2022.950773 (PMC9578668; doi:10.3389/fmicb.2022.950773)
Supplement: Supplementary file 1 [file Table_1.docx]

Table S1. Primers information for 5 DNA sequences

| Name | Length | Direction | Sequence 5ˊ-3ˊ | Reference |
| --- | --- | --- | --- | --- |
| ITS5 | 22 | forward | GGAAGTAAAAGTCGTAACAAGG | White et al. 1990 |
| ITS4 | 20 | reverse | TCCTCCGCTTATTGATATGC |  |
| NS1 | 19 | forward | GTAGTCATATGCTTGTCT C | White et al. 1990 |
| NS4 | 20 | reverse | CTTCCGTCAATTCCTTTAAG |  |
| LROR | 17 | forward | ACCCGCTGAACTTAAGC | Rakotonirainy et al. 1994 |
| LR5 | 17 | reverse | TCCTGAGGGAAACTTCG |  |
| CRPB1 | 20 | forward | CAYCCWGGYTTYATCAAGAA | Castlebury et al. 2004 |
| RPB1Cr | 23 | reverse | CCNGCDATNTCRTTRTCCATRTA |  |
| 983F | 23 | forward | GCYCCYGGHCAYCGTGAYTTYAT | Castlebury et al. 2004 |
| 2218R | 23 | reverse | ATGACACCRACRGCRACRGTYTG |  |
